# Capillary Isoelectric Focusing of Akt Isoforms Identifies Highly Dynamic Phosphorylation in Neuronal Cells and Brain Tissue*

**DOI:** 10.1074/jbc.M115.700138

**Published:** 2016-03-04

**Authors:** Sandra Schrötter, George Leondaritis, Britta J. Eickholt

**Affiliations:** From the Charité-Universitätsmedizin Berlin, Cluster of Excellence NeuroCure and Institute of Biochemistry, Berlin 10117, Germany

**Keywords:** Akt PKB, brain, cell signaling, phosphatase and tensin homolog (PTEN), post-translational modification (PTM), capillary isoelectric focusing, phosphorylation, growth factors, neurons

## Abstract

The PI3K/PTEN/Akt pathway has been established as a core signaling pathway that is crucial for the integration of neurons into neuronal circuits and the maintenance of the architecture and function of neurons in the adult brain. Akt1–3 kinases are specifically activated by two phosphorylation events on residues Thr^308^ and Ser^473^ upon growth factor signaling, which subsequently phosphorylate a vast cohort of downstream targets. However, we still lack a clear understanding of the complexity and regulation of isoform specificity within the PI3K/PTEN/Akt pathway. We utilized a capillary-based isoelectric focusing method to study dynamics of Akt phosphorylation in neuronal cells and the developing brain and identify previously undescribed features of Akt phosphorylation and activation. First, we show that the accumulation of multiple phosphorylation events on Akt forms occur concurrently with Ser^473^ and Thr^308^ phosphorylation upon acute PI3K activation and provide evidence for uncoupling of Ser^473^ and Thr^308^ phosphorylation, as well as differential sensitivities of Akt1 forms upon PI3K inhibition. Second, we detect a transient shift in Akt isoform phosphorylation and activation pattern during early postnatal brain development, at stages corresponding to synapse development and maturation. Third, we show differential sensitivities of Ser^473^-Akt species to PTEN deletion in mature neurons, which suggests inherent differences in the Akt pools that are accessible to growth factors as compared with the pools that are controlled by PTEN. Our study demonstrates the presence of complex phosphorylation events of Akt in a time- and signal-dependent manner in neurons.

## Introduction

Two major signaling components in brain and neuron physiology are the PI3K pathway and its negative regulator, PTEN (tumor suppressor gene phosphatase and tensin homolog deleted on chromosome 10). Among the PI3Ks, the class I group is key in the generation of phosphatidylinositol 3,4,5-trisphosphate (PIP_3_)^4^ by phosphorylation of phosphatidylinositol 4,5-bisphosphate. PIP_3_ mediates the recruitment and subsequent activation of several intracellular kinases, adaptor proteins, and regulators of small GTPases, which is crucial for the propagation of PI3K-dependent signaling. Akt, for example, is a protein kinase promoting cell survival and proliferation by inactivating Bad and glycogen synthase kinase-3 (GSK-3) and induction of mammalian target of rapamycin (mTOR) (1). A body of work indicates that the PI3K signaling pathway is crucial for both the developmental integration of neurons into neuronal circuits and the maintenance of the architecture of individual neurons in the adult brain (2). In fact, PI3K signaling is part of all major steps involved in the neuronal maturation program, including neurite outgrowth, neuronal polarization, axonal branching and synapse formation (2). The activity of class I PI3Ks are antagonized by PTEN, which regulates this signaling pathway by dephosphorylating PIP_3_ to phosphatidylinositol 4,5-bisphosphate (3). As a consequence, the absence of PTEN leads to enhanced PIP_3_ availability, resulting in increased Akt phosphorylation and activation. Interestingly, loss of function mutations of PTEN are among the most common genetic abnormalities in gliomas (4) and also define a spectrum of neurodevelopmental disorders characterized by neurological deficits such as macrocephaly, developmental delay, and mental retardation (5).

The downstream effector Akt is a threonine/serine kinase critical for the regulation of cell growth, survival, proliferation, and differentiation. Deregulation of Akt activity because of aberrant PI3K signaling has been linked to the progression of various pathological conditions including cancer and neurodevelopmental disorders (6, 7). There are three Akt isoforms encoded by three genes: Akt1, Akt2, and Akt3; an alternatively Akt3 splice transcript variant has further been described (8). The exact cellular roles of different isoforms have not been completely established, but loss of individual isoforms can cause different pathologies. The most broadly expressed isoforms, Akt1 and Akt2, demonstrate involvement in controlling survival, growth, and metabolic signaling. For example, Akt1^−/−^ mice show growth retardation and increased apoptosis, and in humans, an overactivation of Akt1 has been associated with Proteus syndrome (9–11). Akt2^−/−^ mice were found to develop diabetes mellitus-like phenotypes (12). Akt3 exhibits the most restricted expression pattern, which is associated with testes and brain, with knock-out mice exhibiting smaller brain sizes (13). In contrast, Akt3 *de novo* germline mutations in humans can cause a spectrum of megalencephaly syndromes (14).

Studies in neurons have identified a large spectrum of Akt functions. During neurodevelopment, for example, Akt has been found to partake in the regulation of neuronal polarization and axon growth with a pool of active Akt found at the axon tip but not the tips of dendrites (15). Neuronal, isoform-specific contributions mediating particular cellular function, on the other hand, are just beginning to emerge. For example, it was demonstrated that depletion of single Akt isoform did not induce significant changes in neuronal polarity, whereas blockage of Akt2 and to a greater extent Akt3 reduced axonal outgrowth responses (7). Nevertheless, comparative studies on the endogenous expression and activity profiles of Akt isoform in neuronal cells and during normal or diseased brain development are largely missing.

Activity of Akt is largely dependent on the phosphorylation status. Although Akt is phosphorylated at numerous site (20–22 phosphorylation sites have been validated, (16)), studies have concentrated mostly on two activating phosphorylation events. The first, Ser(P)^473^, is located in the hydrophobic motif of the protein, and the second, Thr(P)^308^, is located in the catalytic motif (with the numbering of amino acids in accordance of Akt1). Recently, phosphorylation of Ser^477^ and Thr^479^ at the C terminus of Akt1 were shown to promote or even compensate for Ser^473^ phosphorylation (17). A third well studied site is a constitutive, stabilizing phosphorylation at Thr^450^. It is generally accepted that growth factor stimulation leads to the phosphorylation of Akt, which triggers activation of the enzymatic kinase activity. The Ser^473^ and Thr^308^ phosphorylation sites are targeted by different kinases. PDK1 is a PI3K-regulated kinase responsible for phosphorylating Thr^308^, whereas mTORC2 is thought as the main kinase targeting the Ser^473^ Akt site. Mechanisms have been proposed in which phosphorylation at Thr^308^ precedes the Ser^473^ phosphorylation and vice versa (18). In a widely accepted model, Akt interacts with the plasma membrane in a PIP_3_-dependent mechanism, leading to initial phosphorylation of Thr^308^ by PDK1 (19, 20). Interestingly, subsequent phosphorylation of the Ser^473^ site by mTORC2 appears also to be regulated by PIP_3_ (19).

A capillary-based isoelectric focusing (cIEF) method coupled with pan- or phospho-specific antibody-based detection (16, 20–23) had recently been employed to assess the Akt phosphorylation profile in tumor cells and non-neuronal cell lines (16, 20, 21). This method provided sufficient resolution of phospho-specific forms of Akt isoforms under basal, starved, and growth factor-stimulated conditions (16, 21) and permitted the identification of differential Ser^473^ and Thr^308^ phosphorylation events in Akt1 and Akt2 molecules (16). Because of its principle, this cIEF method is ideal to address questions unapproachable by other techniques, including the analyses of differential phosphorylation of Akt isoforms by growth factors or the identification of differential sensitivity to inhibitors (PTEN) or activators (PI3K isoforms) within the PI3K signaling pathway. Here, we further validate cIEF technologies and implement an Akt assay in neuronal cell lines and primary neuronal cultures, as well as in brain tissue at different developmental stages. Our results show a previously undetected shift in Akt isoform phosphorylation/activation pattern during early postnatal brain development and substantial differences in sensitivity of Akt isoforms against growth factors, PI3K inhibition, and PTEN ablation during late stages of neuronal differentiation *in vitro*.

## Experimental Procedures

### 

#### 

##### Cell Culture and Protein Lysates

N1E-115 cells were cultured in DMEM with GlutaMAX, 10% FCS, and 1% penicillin/streptomycin (Invitrogen) and routinely kept at 5% CO_2_ and 37 °C. Wortmannin (WM) treatments (200 nm) were performed at the indicated time points. Primary cortical neurons were dissected from embryonic day 16.5 C57/BL6 mice and cultured on polyornithine-coated coverslips in neurobasal medium (Invitrogen) containing 2% B27 (Life Technologies), 1% penicillin/streptomycin, and 1% GlutaMAX. To control for PKC signal in 21-day *in vitro* (DIV) primary neurons, 80 nm tetradecanoyl-phorbol-acetate (TPA) was directly added to the medium for 24 h. For lysis, N1E-115 and/or primary neurons were washed twice with ice-cold PBS and lysed with Bicine/CHAPS containing 1× aqueous inhibitor and 1× DMSO inhibitor (all cIEF reagents were from Protein Simple). Wistar rat and mouse brains were lysed in RIPA buffer (Sigma-Aldrich) containing protease and phosphatase inhibitors. Lysed proteins were aliquoted and stored at −80 °C. The protein concentration was quantified using the Pierce® BCA protein assay kit (Thermo Scientific). λ-Phosphatase (New England Biolabs) was used according to the manufacturer's protocol. In short, N1E-115 protein samples were incubated with or without phosphatase for 30 min at room temperature directly before performing Western blot or cIEF.

##### Growth Factor Stimulation

For growth factor stimulation experiments, N1E-115 cells were seeded in 6-well plates. After starvation in DMEM without serum for 48 h, the cells were stimulated with 100 nm insulin. Primary cortical neurons were plated in 12-well plates and starved for 2 h prior to growth factor treatment. BDNF was applied at 50 ng/ml and EGF was applied at 40 ng/ml for 15 min.

##### Viral Infection of Primary Cortical Neurons

For viral transduction, a modified lentiviral vector was used in which a human Synapsin-1 promoter drives the expression of the RFP-Cre transgene. Lentiviruses were produced by co-transfecting HEK293T cells with the lentiviral vector and two helper vectors, pVSVg and pCMV-delta R8.9 (24, 25). Viral supernatants were collected 48 h after transfection, and virus particles were added to cultured floxed PTEN neurons 12 days after plating (12 DIV). At 9 days post-transduction, neurons (21 DIV) were harvested in Bicine/CHAPS buffer with freshly added inhibitors (Protein Simple), and lysates were prepared for Western blotting and cIEF.

##### NanoPro100^TM^ Assay

A master mix of (5–8) nested G_2_ premix (Protein Simple), pI standard ladder 3 (Protein Simple), and an additional 5.5 pI standard was prepared. According to a final protein concentration of 75–125 ng/capillary, sample diluent (Protein Simple), and DMSO inhibitor (Protein Simple), and then the protein sample were mixed. The following primary antibodies were used at dilutions of 1:25–1:50: pan-Akt (Cell Signaling, catalog no. 9272), Akt1 (Millipore, catalog no. 05-669), Akt2 (Cell Signaling, catalog no. 3063), Akt3 (Upstate, catalog no. 03-383), Thr(P)^308^-Akt (Cell Signaling, catalog no. 2965), Ser(P)^473^-Akt (Cell Signaling, catalog no. 4060), and Thr(P)^450^-Akt (Cell Signaling, catalog no. 9267) (see Fig. 3). Bound primary antibodies were detected with HRP goat anti-rabbit secondary antibody (Protein Simple) at 1:100 dilutions. Samples and antibodies were transferred to the assay plate. Luminol/peroxide, washing buffer, catholyte, and anolyte (all Protein Simple) were used according to the manufacturer's protocol. Akt isoforms were separated by isoelectric focusing for 40 min at 21,000 μW, followed by immobilization through UV exposure for 100 s. Primary antibodies were incubated in the capillary for 4 h with two subsequent wash steps of 150 s each. The secondary antibody was incubated in the capillary for 1 h with two subsequent wash steps of 150 s each. Last, luminol/peroxide reagent was passed through the capillaries, and chemiluminescence was detected. Prior analyses of the optimal linear detection range identified 240 s of exposure as optimal, which was used in all cIEF experiments. Peak integration and pI marker calibration was performed using Compass^TM^ software as previously described (26). All cIEF graphs show the representative profile of one experimental condition. All experiments were undertaken in two independent biological replicates, each consisting of two technical replicates unless stated differently in the figure legend.

##### Western Blot Analysis

Protein lysates were prepared with 4× Roti-Load (5 μg of total protein), loaded on 8% SDS gels, stacked at 60 V for 30 min, and separated at 120 V for 60 min. Proteins were transferred to nitrocellulose membrane using a wet blot tank system (Bio-Rad) for 2 h. The membranes were then blocked for 30 min at room temperature with 5% skim milk before incubating with the primary antibodies. Primary antibodies were prepared at a dilution of 1:1000 in 5% skim milk and incubated overnight at 4 °C. Following incubation, membranes were washed three times with TBS-T at room temperature for 5 min. Secondary antibodies were prepared at a dilution of 1:3000 in 5% skim milk. The membranes were incubated for 30 min at room temperature. Following secondary antibody incubation, membranes were washed three times with TBS-T. Membranes were imaged using the Fusion SL system from Vilber Lourmat.

## Results

### 

#### 

##### Akt cIEF Assay Development and Peak Identification

**FIGURE 1.**
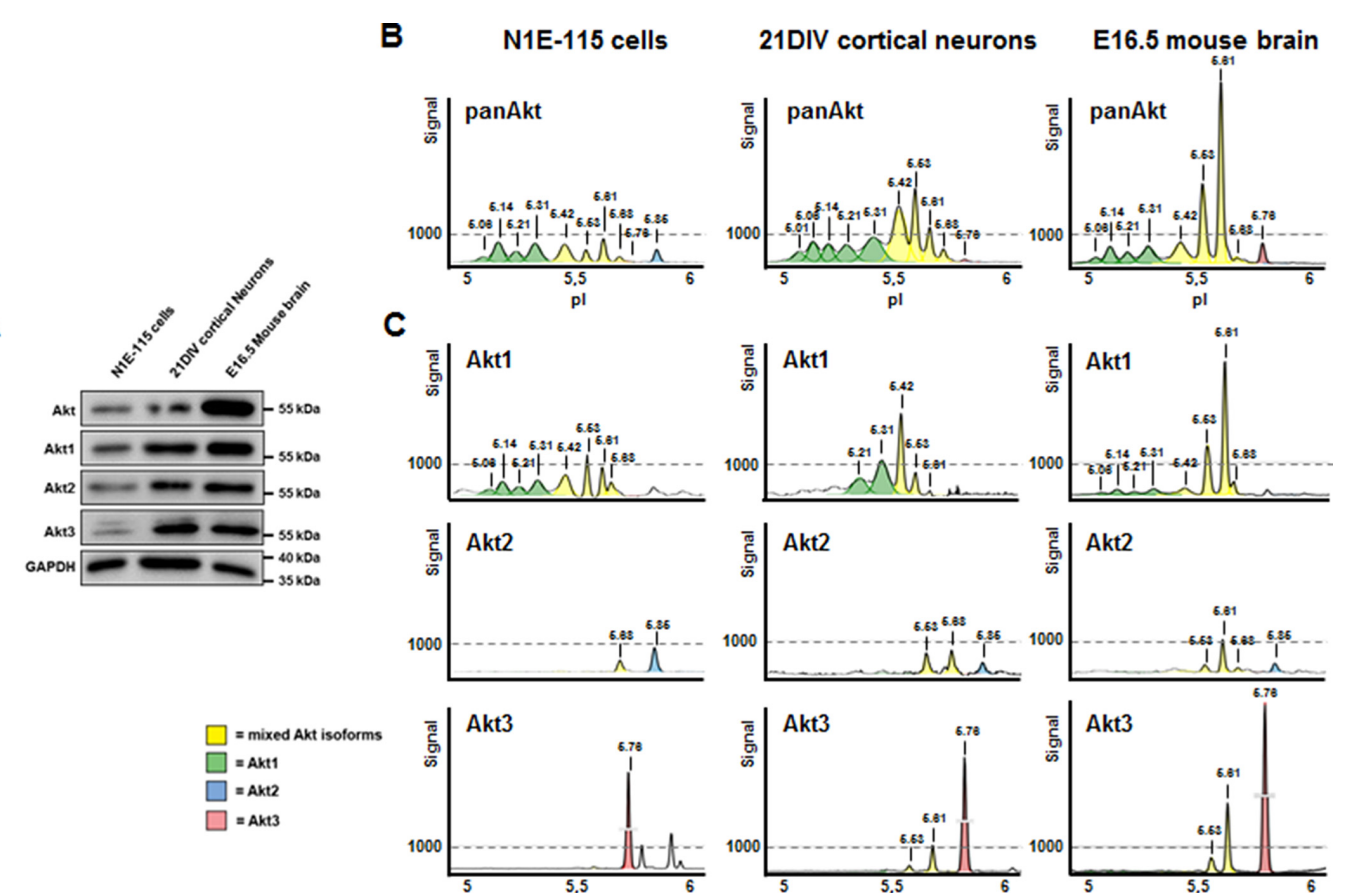
**Akt cIEF assay development and peak identification.** *A*, Western blot analysis of Akt isoforms (Akt1–3) in cell lysates obtained from N1E-115 neuroblastoma cells, primary cortical neurons (*21DIV*), and embryonic day 16.5 (*E16.5*) mouse brain. *B*, cIEF analysis of the cell lysates using a pan-Akt antibody shows a regular Akt profile with 9 or 10 conspicuous peaks that are separated according to protein charge distribution. *C*, cIEF analysis of cell lysates using isoform-specific Akt antibodies. In cIEF profiles, Akt1 peaks are colored in *green*, Akt2 peaks are in *blue*, and Akt3 peaks are in *red*. Peaks with mixed Akt isoforms are colored in *yellow*.

To investigate Akt isoforms and their post-translational modifications (PTMs) in a neuronal context, we used N1E-115 neuroblastoma cells, primary cortical neurons, and whole brain lysates obtained from embryonic day 16.5 mice. In all lysates, Western blotting (WB) identified the expression of the three Akt isoforms Akt1–3, with the main form being Akt1 (Fig. 1*A*). We used the same lysates on the nanocapillary immunoassay technology that is based on isoelectric focusing (cIEF). In this technique, protein separation occurs according to net charge, allowing the separation and detection of phospho-forms, as well as isoforms of the same protein by a single antibody. We probed N1E-115 cell lysate with a pan-Akt antibody and identified 10 peaks in the cIEF profile, with corresponding isoelectric points (pIs) of 5.06, 5.14, 5.21, 5.31, 5.42, 5.53, 5.61, 5.68, 5.76, and 5.85 (Fig. 1*B*). In accordance with previous reports, the pIs of all Akt isoforms lie between 5.0 and 6.0 (21). In cell lysates of primary cortical neurons, pan-Akt detected the same 10 peaks and additionally a more acidic peak species with a pI of 5.01. In brain lysate, on the other hand, all of the N1E-115 cell-specific peaks were detected, with the exception of the 5.85 peak. These results demonstrate that cIEF can be used in neuronal cells and brain tissue to obtain unique and reproducible neuronal AKT peak profiles using a single detection reagent. Across the different neuronal samples tested, the relative abundance of Akt molecules changed significantly, indicating potential context-dependent modifications or cell/tissue-specific alterations in isoform expression profiles.

To characterize neuronal peak profiles further, we used the isoform-specific antibodies recognizing Akt1, Akt2, or Akt3 (Fig. 1*C*). In N1E-115 cells, the Akt1 antibody recognized the most acidic peaks: 5.06, 5.14, 5.21, 5.31, 5.42, 5.53, 5.61, and 5.68, with 5.53 showing the largest signal. The Akt2 antibody gave a more restricted panel with two conspicuous peaks at pIs of 5.68 and 5.85. In this case, the largest peak was present at pI 5.85. Detection using the Akt3 antibody gave two specific peaks with pIs of 5.61 and 5.76, with 5.76 being the dominant signal. In addition, three nonspecific peaks with a pI of >5.80 were found, which may represent the upper band detected by WB of N1E-115 cell lysate (Fig. 1*A*). Primary cortical neurons and brain lysate showed similar peak profiles, except that the Akt2 antibody recognized an additional peak at 5.61. In all three neuronal lysates, Akt1 exhibited slight changes in the specific pI across the samples. The main Akt1 molecule was found in neuroblastoma cells at pI 5.53, in cortical neurons at 5.42, and in brain lysate at 5.61. Thus, the extent of PTMs seems to vary between different cell types. A summary of all peaks with the corresponding antibodies is shown in Fig. 3*A*.

**FIGURE 2.**
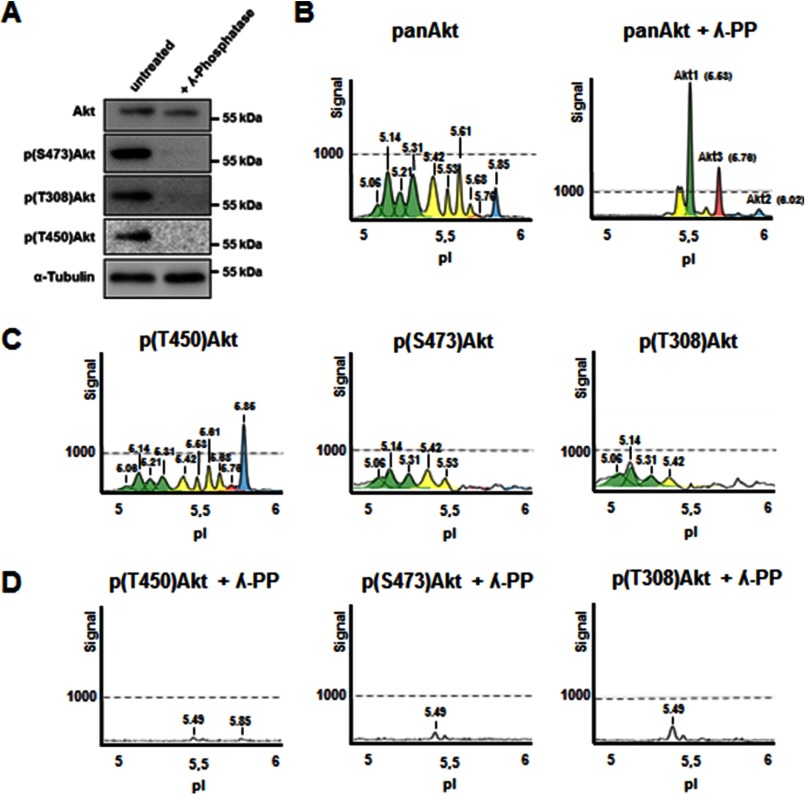
**Identification of phospho-specific Akt peaks in N1E-115 neuroblastoma cells.** *A*, N1E-115 cell lysates were treated with λ-phosphatase before Western blot analyses using indicated antibodies. *B*, the same cell lysates were analyzed using cIEF; peak profiles demonstrate the loss of peaks <pI 5.49 after λ-phosphatase treatment, identifying them as phosphorylation-containing peaks. *C*, NIE-115 cell lysates were analyzed by cIEF using the indicated antibodies. *D*, after λ-phosphatase treatment of NIE-115 cell lysates, all peaks of <5.49 in the Akt cIEF profile are lost, identifying them as phosphorylation-containing peaks. The three phosphorylation-specific antibodies only detected minor peaks, confirming the specificity of cIEF in detecting phosphorylated Akt species.

**FIGURE 3.**
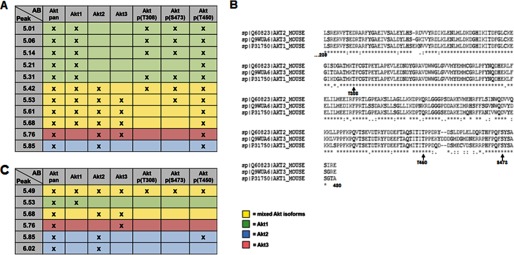
**Identified Akt peaks and alignment of Akt phosphorylation sites studied.** *A*, table showing identified cIEF peaks using different Akt antibodies in cell lysates obtained from neuronal cells. *B*, sequence alignment of the major phosphosites of the three Akt 1–3 isoforms: Thr(P)^308^, Thr(P)^450^, and Ser(P)^473^ (assignment of P sites is based on Akt1). *C*, table showing identified cIEF peaks using different Akt antibodies in NIE cell lysates treated with λ-phosphatase. In both tables, Akt1 specific peaks are colored in *green*, Akt2 peaks are in *blue*, and Akt3 peaks are in *red*. Peaks with mixed Akt isoforms are colored in *yellow*.

To unequivocally assign peaks to their phospho-states, we removed phosphate groups in lysates with λ-phosphatase. WB analyses using Ser(P)^473^-, Thr(P)^308^-, and Thr(P)^450^-Akt antibodies confirmed the specificity of the treatment (Fig. 2*A*). When compared with cIEF profiles of untreated control cell lysates, λ-phosphatase resulted in the absence of cIEF peaks between pI 5.06 and 5.42, identifying these as phosphorylated Akt forms (Fig. 2*B*). In parallel, the peaks at pI 5.53 and 5.76 showed the greatest increase, with the appearance of a new peak at 6.02, which categorized these signals as the three nonphosphorylated Akt isoforms (Fig. 2*B*). The summary of antibodies recognizing Akt specific peaks after dephosphorylation by λ-phosphatase can be found in Fig. 3*C*. To confirm this result, we also used the phospho-specific antibodies Ser(P)^473^, Thr(P)^308^, or Thr(P)^450^ in cIEF. Thr(P)^450^ has previously been characterized as a constitutive Akt phosphorylation event, and indeed, detection using the anti-Thr(P)^450^-Akt antibody in cIEF induced the exact same peak profile as the pan-Akt antibody (Fig. 2*C*, *left panel*). However, in case of Thr(P)^450^-Akt, λ-phosphatase treatment removed almost all peaks detected, indicating the specificity of the antibody toward phosphorylated Akt species (Fig. 2*D*, *left panel*). Interestingly, when compared with the pan-Akt profile, the peak detected with Thr(P)^450^ at pI 5.85 was greatly increased. This may suggest a higher level of Thr^450^ phosphorylation for Akt2 and/or differential affinity of the pan-Akt/Thr(P)^450^ antibodies to Akt1 and Akt2 isoforms (Fig. 2*B* and *C*). The results for the Akt3-specific antibody and the phosphatase treatment indicate that nonphosphorylated Akt3 peaks at 5.76. This peak is also detected with the Thr^450^ antibody, suggesting a heterogeneous peak most likely consisting largely of nonphosphorylated Akt3, with a minor population of phospho-Akt2 molecules, only found after specific growth factor stimulation (data not shown). Thus, for the first time, we were able to identify the unphosphorylated Akt3 isoform with cIEF in neuronal cells. The pI of murine Akt3 with 5.76 is slightly higher than the theoretical prediction of 5.71.

Antibodies of the two activating phosphorylation sites Thr^308^ and Ser^473^ recognized, as expected, the most acidic peaks of the cIEF profile. Thr^308^ antibody detected four peaks in untreated N1E-115 cells (5.06, 5.14, 5.31, and 5.42). The same four peaks and an additional peak were found with the Ser^473^ antibody (5.06, 5.14, 5.31, 5.42, and 5.53) (Fig. 2*C*). Because one peak recognized by Ser(P)^473^ is not recognized by Thr^308^, our results show that, at least under basal conditions, the two activating phosphorylation sites can occur independent of each other. Furthermore, we conclude that the five most acidic peaks must vary in their modifications by other phosphorylation sites or other post-translational modifications (*i.e.* ubiquitination, sumoylation). Previous studies reported the presence of up to 22 validated Akt phosphorylation sites (16). We tested commercially available antibodies for the known phosphorylation sites Thr^34^ and Tyr^326^ but were not able to obtain specific signals with either WB or cIEF. Overall, our results are in good agreement with a recent study of Akt by cIEF in human cancer cell lines (16).

##### Dynamics of Akt Phosphorylation in N1E-115 Cells

**FIGURE 4.**
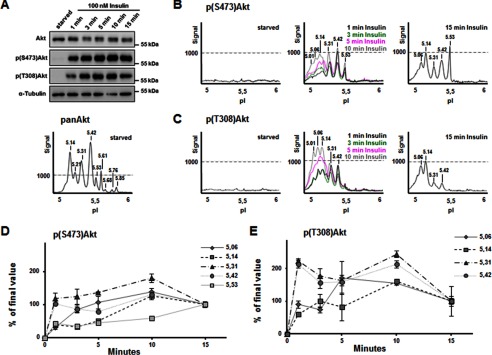
**Dynamics of Akt phosphorylation in response to insulin in N1E-115 cells.** *A*, N1E-115 cells were starved for 48 h before insulin stimulation for different times (1–15 min). Cell lysates were analyzed by Western blotting using indicated antibodies. *B* and *C*, NIE-115 cell lysates were analyzed in parallel by cIEF using Ser(P)^473^-Akt antibody (*B*) and Thr(P)^308^-Akt antibody (*C*). *D* and *E*, the area under each peak was quantified for Ser(P)^473^-Akt (*D*) and the Thr(P)^308^-Akt antibody (*E*), demonstrating the phosphorylation dynamics in time of individual peaks.

Akt is recruited to the plasma membrane by interaction with its phosphoinositide docking sites, following stimulation of PI3K in response to various growth factors. To get further insight into the influence of PI3K signaling on Akt phosphorylation, we stimulated N1E-115 cells with 100 nm insulin over a time course of 15 min. Prior stimulation, the cells were starved for 48 h in serum-free medium to erase baseline phosphorylation. As shown by WB, no Thr^308^ or Ser^473^ phosphorylation was detectable using this starvation protocol (Fig. 4*A*), which was confirmed by testing cell lysates also with the two phospho-specific antibodies Thr(P)^308^ or Ser(P)^473^ in cIEF (Fig. 4, *B* and *C*, *left panels*). For these experiments, the pan-Akt antibody was used to ensure equal loading in cIEF samples (Fig. 4*A*, *lower panel*). Following insulin stimulation, robust Akt phosphorylation was already detectable after 1 min with both phospho-antibodies in WB and cIEF (Fig. 4, *A–C*). Remarkably, although detection of Akt phosphorylation upon acute insulin treatment by WB revealed little insight into the phosphorylation dynamics (Fig. 4*A*), the resulting cIEF profiles unraveled unique Akt phosphorylation features, because phosphorylation on Ser^473^ and Thr^308^ appeared to occur concomitantly in a cooperative process in time. Peak increases were first observed in the less acidic peaks 5.42 and 5.31. At later time points, there was a gradual appearance and increase of more acidic and often poorly resolved peaks with pIs of 5.06–5.14. This result suggests that the accumulation of multiple and distinct phosphorylation events on Akt forms occurs concurrently with Ser^473^ and Thr^308^ phosphorylation. After 15 min of insulin stimulation, four prominent acidic peaks were detected for both Ser(P)^473^ and Thr(P)^308^ (with pIs of 5.06, 5.14, 5.31, and 5.42), as well as an additional one in case of Ser(P)^473^ (pI of 5.53) (Fig. 4, *B* and *C*, *right panels*). The latter peak was only faintly detected by the Thr(P)^308^ antibody and most likely corresponds to the Ser(P)^473^ Akt form identified in N1E-115 lysates under basal conditions (Fig. 2). It has to be noted that the 5.21 peak detected with the pan-Akt antibody was not detected with neither the Ser(P)^473^ nor the Thr(P)^308^ antibody. However, because Thr(P)^450^ detected the 5.21 peak (Fig. 2) and because it was sensitive to treatment with λ-phosphatase (Fig. 2), we surmise that this peak may represent forms of Akt precursors for Ser^473^ or Thr^308^ phosphorylation.

Quantification of the area of single peaks at each time point revealed that maximal signals were reached after 10 min of insulin treatment, followed by small decreases at 15 min (Fig. 4, *D* and *E*). The only peak with a steady increase over the entire time course was the 5.53 peak with the Ser^473^ antibody (Fig. 4*D*). Comparison of the of Ser^473^ and Thr^308^ peaks dynamics in time identified differences and similarities of specific Akt phosphorylation forms. For example, the peak previously identified as Akt1 (pI 5.06) showed a steady increase during the course of the first 5 min of insulin treatment followed by a small decrease over the next 10 min of incubation, when monitored with the Ser(P)^473^ antibody (Fig. 4*D*). In contrast, when monitored with Thr(P)^308^, increases of this peak during the 15-min insulin stimulation showed a relatively unstable development (Fig. 4*E*). Similarly, the peaks previously identified to contain Akt1/Akt2 (pI 5.42) showed overlapping patterns for both phospho-specific antibodies over the time course of stimulation, characterized by a rapid increase to maximal levels at 10 min of treatment (Fig. 4, *D* and *E*). It should be noted here that we cannot exclude the possibility that the peaks identified by cIEF are heterogeneous with respect to Ser(P)^473^ and Thr(P)^308^, as well additional phosphorylation or other modifications. Given the complexity of growth factor-induced Akt phosphorylation cIEF profiles, a certain level of heterogeneity should be expected. Nevertheless, our results are in line with previous reports and support that both Ser^473^ and Thr^308^ phosphorylation events can occur uncoupled of each other during growth factor stimulation.

##### Dynamics of Akt Dephosphorylation in N1E-115 Cells

**FIGURE 5.**
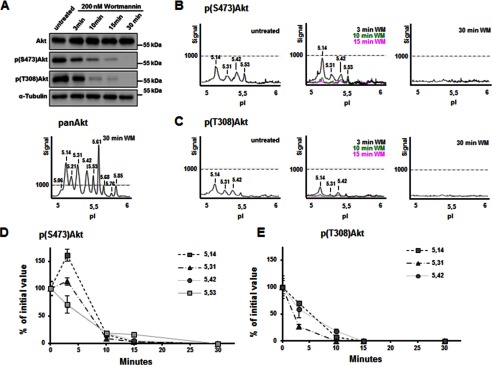
**Dynamics of wortmannin-induced Akt dephosphorylation in N1E-115 cells.** *A*, N1E-115 cells were treated with the PI3K inhibitor wortmannin at 200 nm for different periods of time (3–30 min) before Western blot analysis using indicated antibodies. *B* and *C*, cell lysates were analyzed in parallel using cIEF with Ser(P)^473^-Akt (*B*) and Thr(P)^308^-Akt (*C*). *D* and *E*, the area under each peak was quantified for Ser(P)^473^-Akt (*D*) and the Thr(P)^308^-Akt (*E*) signals.

Because the cIEF approach provided unique insights into the dynamics of growth factor-stimulated Akt phosphorylation in NIE-115 cells, we next assessed the effects of acute PI3K inhibition on Akt phosphorylation. We tested different concentrations of WM, a general inhibitor of PI3K, and found significant loss of Akt phosphorylation at Thr^308^ and Ser^473^ when using 200 nm WM (data not shown). WB confirmed the gradual decrease in Ser(P)^473^ and Thr(P)^308^ over time, with an apparent complete removal of Akt phosphorylation after 30 min of WM incubation (Fig. 5*A*; the *lower panel* shows a typical pan-Akt cIEF profile in this sample). This result was confirmed by cIEF, which detected no signals in the peak profiles of Ser(P)^473^ and Thr(P)^308^ at 30 min of WM treatment (Fig. 5, *B* and *C*, *right panels*). Analyses of Akt dephosphorylation in response to inhibition of PI3K in time identified differential progression of events. It was evident, for example, that in comparison to Ser(P)^473^, the Thr(P)^308^ site seems to be more sensitive to WM treatment in cIEF analyses (Fig. 5*A*). Upon short WM treatment, the cIEF peak profiles revealed unexpected features, for example, it detected a specific and transient increase in Ser^473^ phosphorylation in the identified Akt1 peak at pI 5.14 (Fig. 5, *B* and *D*). Nevertheless, this effect was specific for the peak with pI 5.14 (and to some extent also for the 5.31 peak), but not for other Akt1/Akt2 peaks that showed similar patterns for both Ser^473^ and Thr^308^ dephosphorylation (Fig. 5, *B–E*). We obtained similar results with primary cortical neurons (data not shown). In all experiments, we did not observe a substantial and statistically significant net increase of total Ser^473^ phosphorylation upon acute (2–3 min) PI3K inhibition (Fig. 5*A*). Thus, in principle, it is likely that this apparent increase of the Ser^473^ Akt1 5.14 form may result from acute gain or loss of other PMTs from other Akt1 forms. Nevertheless, these data suggest that at least for some Akt forms, acute loss of PIP_3_ results in transient up-regulation of Ser(P)^473^ but not Thr^308^.

Interestingly, when we compared the cIEF profiles of pan-Akt after 48 h of starvation and 30 min of WM treatment, which were used as loading controls (Figs. 4*A* and 5*A*), we saw a different pattern. The Akt profile of WM-treated N1E-155 cells was more similar to the Akt profile of untreated N1E-115 cells, when compared with the profile of starved N1E-115 cells. This result may indicate that starvation and the limitation of PIP_3_ availability through PI3K inhibition have an effect on different Akt phosphorylation events. Alternatively, other PTMs may play a role in controlling Akt activity.

##### Dynamics of Akt Phosphorylation during Postnatal Rat Brain Development

**FIGURE 6.**
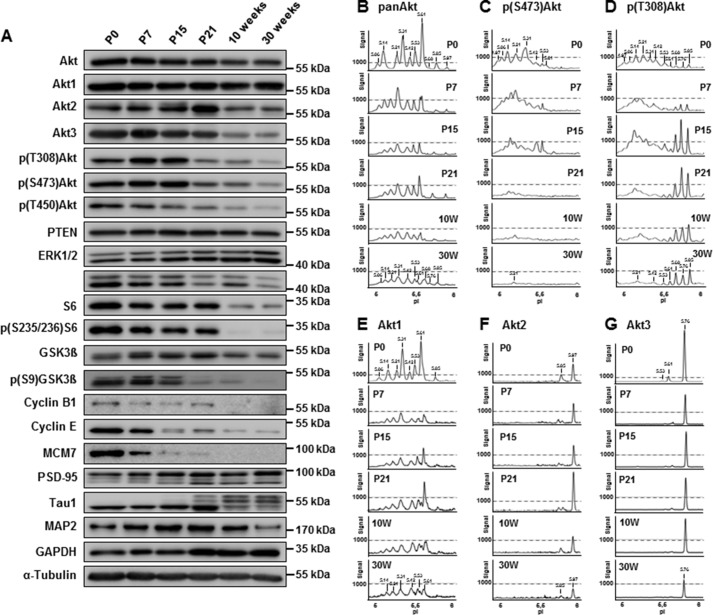
**Analysis of PI3K signaling strength during rat brain development.** *A*, Western blot analysis of rat brain lysates obtained at different postnatal and adult stages (P0 to 30 weeks) using antibodies against structural and signaling proteins related to neuronal development, including components of the PI3K/PTEN and ERK signaling pathways. *B–G*, the same lysates were analyzed by cIEF using pan-Akt (*B*), Ser(P)^473^-Akt (*C*), or Thr(P)^308^-Akt (*D*) or the isoform-specific antibodies recognizing Akt1 (*E*), Akt2 (*F*), or Akt3 (*G*). The most prominent changes in Akt peak distribution during brain development and maturation occur in the Akt1 isoform.

Despite the widely accepted importance of the PI3K/Akt pathway to neuronal development (2), there is currently little knowledge concerning the endogenous profile and regulation of Akt phosphorylation during normal brain development. Therefore, we analyzed whole brain extracts obtained from rat brains during postnatal (P0, P7, P15, and P21) and adult stages (10 and 30 weeks) for the expression profile of Akt phosphorylated forms. Samples were first characterized by WB with a panel of antibodies against structural and signaling proteins related to neuronal development, including components of the PI3K/PTEN and ERK pathways (Fig. 6*A*). In general, the expression and/or phosphorylation levels of a number of signaling proteins analyzed were down-regulated either shortly before (at P15–P21; pERK, pGSK3β, Thr(P)^308^, Ser(P)^473^, or Thr(P)^450^-Akt) or just after hard wiring was completed (after P21; S6, pS6, or GSK3β). A general down-regulation during postnatal and adult stages was also found for the anti-pan-Akt antibody. On the other hand, whereas the amount of Akt1 seemed to remain stable for all the different ages tested, Akt2 and Akt3 protein levels show either steady increases in expression (Akt2) or remain high (Akt3) during postnatal development until P21, before sharply decreasing to lower expression levels.

**FIGURE 7.**
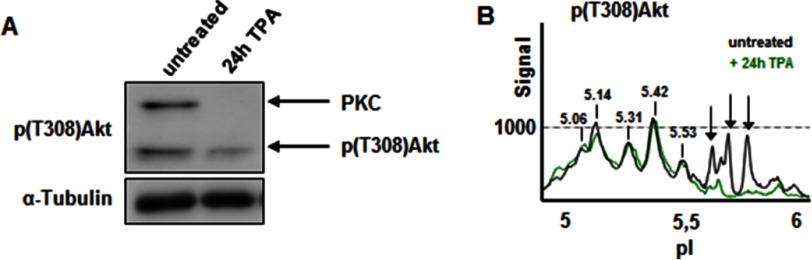
**Unspecific detection of PKC with the Thr(P)^308^-Akt antibody in neuronal cells.** *A*, cell lysates obtained from nontreated or TPA-treated 21 DIV cortical neurons were analyzed by Western blotting. Thr(P)^308^-Akt antibody detection identified phosphorylated Akt, as well as a slower migrating Thr(P)^308^-immunoreactive band of approximately 70 kDa. This band was sensitive to PKC inhibition with long term TPA treatment for 24 h. Thr(P)^308^-Akt was not affected. *B*, the three additional peaks in the cIEF profile recognized by Thr^308^ but not Ser^473^ (pI 5.68, 5.76, and 5.85) were not detected after TPA treatment.

When the brain samples were analyzed by cIEF, prominent shifts in the peak profile occurred throughout postnatal development for pan-Akt, Akt1, and Ser(P)^473^-Akt (Fig. 6, *B–E*), whereas the profiles for Akt2 and Akt3 remained largely unchanged (Fig. 6, *F* and *G*). The cIEF profile of Thr(P)^308^-Akt demonstrated dynamic changes similar to that of the Ser(P)^473^-Akt profile, with a transient increase in highly acidic peaks at P7/P15. However, specific to the Thr(P)^308^ profile was a conspicuous and progressive up-regulation of basic peaks during postnatal development (peaks with pIs of 5.68, 5.76, and 5.85; Fig. 6*D*); only some of these peaks overlapped with those recognized by Akt1, Akt2, and Akt3 antibodies. The appearance of these peaks correlated strongly with up-regulation of a ∼70-kDa Thr(P)^308^-positive band in WB. Following characterization of this Thr(P)^308^ band by mass spectrometry, we established that, in actual fact, it corresponded to phosphorylated forms of classic PKCs (data not shown). To confirm these results, we used a 24-h TPA treatment to down-regulate classic PKCs and prevent its phosphorylation. Indeed, we found a loss of the slow migrating band of ∼70 kDa in WB after TPA treatment (Fig. 7*A*). When analyzing these samples with cIEF, the basic, nonspecific Thr(P)^308^-Akt peaks were not detected anymore (Fig. 7*B*). In conclusion, the identified cIEF peak shift to more acidic Akt forms that occurred during postnatal development signifies altered phosphorylation during stages corresponding to synapse development and maturation.

##### Differential Regulation of Akt Phosphorylation by PTEN and Growth Factors in Primary Neurons

Numerous studies have highlighted the importance of the PI3K/PTEN/Akt pathway during almost all major stages of the neuronal maturation program, including neurite outgrowth, neuronal polarization, axonal branching, and synapse formation (2, 27–29). Activation of this pathway primarily depends on growth factors like BDNF, insulin, and IGF-1 (30, 31). In some pathological conditions, activation of the pathway can also be driven by inhibition of PTEN through deletion or inactivating mutations (32). It is still unanswered whether these two approaches result in the same pattern of Akt phosphorylation. We took advantage of the cIEF Akt assay and primary cortical neuron cultures established from PTEN^fl/fl^ mice to address this question.

**FIGURE 8.**
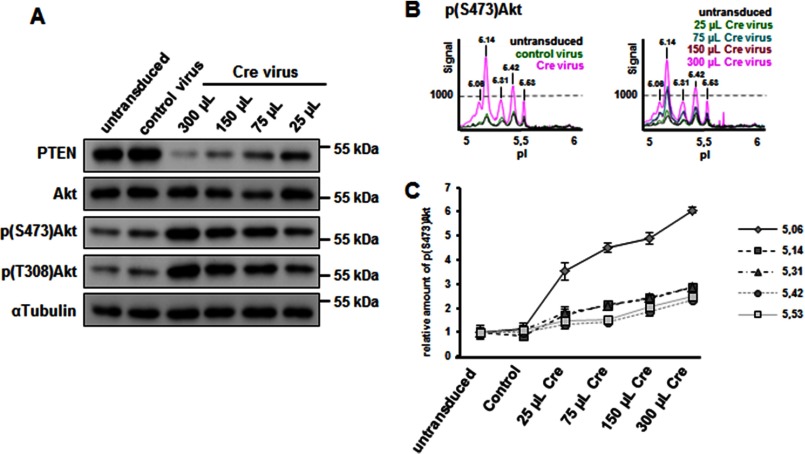
**Effect of gradual PTEN loss on Akt phosphorylation in primary neurons.** *A*, cortical neurons obtained from PTEN^fl/fl^ mice were infected at 12 DIV with control virus or Cre virus, before cell lyses and Western blot analyses at 21 DIV. Increasing concentrations of Cre virus leads to a gradual loss of PTEN and a concomitant increase in Akt phosphorylation at Ser^473^ and Thr^308^. *B*, cell lysates were analyzed in parallel by cIEF using Ser(P)^473^-Akt. The peak profiles demonstrate that PTEN loss increases mostly the most acidic Akt (Akt1) peaks. *C*, the area under each peak from three independent experiments, each analyzed for two technical replicates, was quantified for Ser(P)^473^-Akt. At the highest concentration used, Cre-induced PTEN-loss led to a 6× increase in Ser^473^ phosphorylation of Akt1 (pI 5.06).

PTEN^fl/fl^ neuron cultures (at 12 DIV) were either left untreated or were infected with a control RFP lentivirus or with increasing amounts of a Cre lentivirus. By using this approach, we achieved a linear range in the reduction of PTEN levels in neurons at 21 DIV, as detected by WB (Fig. 8*A*). In accordance, Akt phosphorylation on Ser^473^ and Thr^308^ were both up-regulated (Fig. 8*A*), which we also found in cIEF profiles (Fig. 8*B*). The cIEF peak profiles of lysates obtained from cortical neurons were identical to the profiles detected in lysates of N1E-115 (Figs. 2, 4, and 5). As shown in Fig. 8*B*, all Ser^473^ peaks were responsive to PTEN depletion. Quantification of peak areas against PTEN levels showed also a similar pattern of up-regulation for all Akt Ser^473^ peaks, although the 5.06 and 5.14 peaks appeared substantially more sensitive to PTEN depletion (Fig. 8*C*). This experiment shows the complete profile of Akt Ser^473^ forms that can be achieved by release of PTEN-dependent dephosphorylation of PIP_3_ in cortical neurons.

**FIGURE 9.**
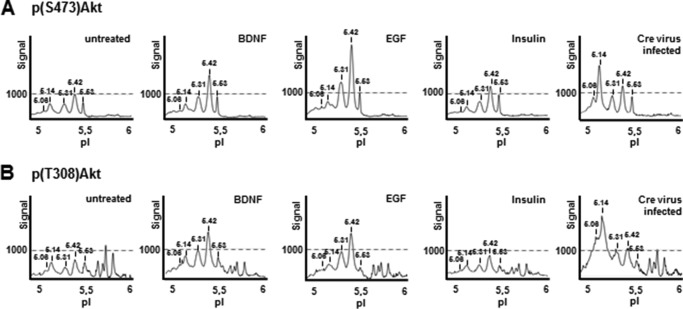
**Context-dependent Akt phosphorylation signatures in cortical neurons.** 21-DIV cortical neurons were stimulated with BDNF, EGF, or insulin for 15 min. In parallel, cortical neurons obtained from PTEN^fl/fl^ mice were infected at 12 DIV with Cre virus and cultured until 21 DIV. All cell lysates were analyzed by cIEF with Ser(P)^473^-Akt antibody (*A*) and Thr(P)^308^-Akt antibody (*B*). Although Akt peak distributions in response to growth factor treatment are largely identical, PTEN loss induces a strong increase in the most acidic Akt1 phosphorylated peaks.

We then compared the PTEN-controlled Akt profile with the one that is accessible to growth factor-induced activation of PI3K under similar culture conditions. We treated 21-DIV cortical neurons with BDNF, insulin, and EGF for 15 min. As shown in Fig. 9, although all three growth factors and PTEN depletion induced comparable Akt phosphorylation detected by WB (not shown), analysis of samples by cIEF showed striking differences between the changes in Ser(P)^473^-Akt in response to growth factors or PTEN depletion (Fig. 9*A*). Whereas the less acidic forms with pIs 5.42 and 5.53 appeared to be more sensitive to all growth factors tested, the acidic Akt forms were more sensitive to PTEN depletion (Fig. 9*A*). Importantly, a similar trend was observed with the Thr^308^ peaks (Fig. 9*B*). Our results suggest inherent differences in the Akt pools (in terms of PTM, in particular phosphorylation) that are accessible to growth factors as compared with the pools that are controlled by availability of PIP_3_ *per se*, at least in mature primary neurons. We believe that this might have important implications for downstream signaling that have to date been unappreciated.

## Discussion

The aim of this study was to understand the activation of Akt isoforms in more detail and in a neuronal background, because previous studies have focused on cancer cell lines or tumor tissue (16, 20–23). We performed a series of growth factor stimulation, PI3K inhibition, and PTEN deletion experiments and assessed the impact on the phosphorylation states of Akt using the Thr^308^ and Ser^473^ phospho-specific antibodies. The advantage of these antibodies is that they were generated against a specific phosphosite of the protein, which is conserved in all three isoforms; therefore they do not favor one or the other Akt isoform. In addition, compared with approaches utilizing a pan-Akt antibody (16, 20), use of phospho-specific antibodies allowed us to assess the correlation and the temporal dynamics and patterns of these activation-specific phosphorylation events. Using cIEF, we were able to unambiguously resolve 4–5 major Akt peaks positive for the activating phosphorylation at Thr^308^ and Ser^473^. Virtually the same peaks with pI values of 5.06, 5.14, 5.31, 5.42, and 5.53 were identified independently in neuroblastoma (Figs. 2, 4, and 5) and primary neurons (Figs. 8 and 9), under various culture conditions and treatments. Interestingly, a slightly different pattern emerges in P0 rat brain tissue, where additional peaks with pI 4.97 and 5.21 appeared (Fig. 6). A detailed characterization based on Akt isoform antibodies and phosphatase experiments in neuroblastoma cells (Figs. 1 and 2) suggested that the peaks with pI 5.06, 5.14, and 5.31 correspond primarily to Akt1, whereas peaks with pI 5.42, and 5.53 correspond to mixed Akt isoforms, most likely Akt1 and Akt2 (Fig. 3).

In addition to Akt isoform identity, it should be noted that inspection of the cIEF profile acute changes during insulin-induced Akt phosphorylation (Fig. 4) suggests further degrees of heterogeneity. Some peaks were poorly resolved or appeared with small shoulder peaks, and this was more evident for the most acidic Ser^473^/Thr^308^-phosphorylated Akt forms (pI 5.06, 5.14, and 5.31). A similar situation was observed for Akt1 phosphorylation in insulin-treated HCT116 colon cancer cells (16). This highlights the fact that the Ser^473^/Thr^308^-phosphorylated Akt forms apparently are further differentiated with respect to additional phosphorylation (and/or other post-translational) modifications. One can only surmise that these distinct Ser^473^/Thr^308^-phosphorylated Akt molecules may differ in their engagement into substrate recognition and phosphorylation of the numerous Akt substrates *in vivo*. In addition, we noticed a difference in the Akt profile of starved and WM-treated neuroblastoma cells. Because it has previously been shown that other PTMs—for example ubiquitination (33), sumoylation (34), or *O-*GlcNAcylation (35)—can influence the activity of Akt, we hypothesize that co-dependences of PTMs may contribute to the control of Akt regulation in situations of general limitation of nutrients and reduced PIP_3_ levels following PI3K inhibition.

Through analyses of the acute changes of Thr^308^- and Ser^473^-phosphorylated Akt forms during insulin or Wortmannin treatments, we were able to provide strong evidence for an uncoupling of Ser^473^ from Thr^308^ Akt phosphorylation. First, at least one peak recognized by Ser(P)^473^ was not recognized by Thr^308^ in insulin-treated neuroblastoma cells (Figs. 2 and 4). Second, comparison of Ser^473^ and Thr^308^ peaks during insulin or wortmannin treatments revealed additional differences in dynamics of phosphorylation or dephosphorylation of specific Akt forms, respectively. For example, Akt1 identified peaks showed distinct temporal patterns during insulin (pI 5.06) and wortmannin (pI 5.14) treatments. These results, together with results from previous recent studies (16), suggest a significant level of uncoupling of Ser^473^ and Thr^308^ Akt phosphorylation events. This uncoupling may well relate to different Akt isoforms as suggested by Guo *et al.* (16) but also in response to growth factor activation or PI3K inhibition as indicated in this study. Indeed, we obtained paradoxical results following acute inhibition of PI3K by wortmannin. These experiments demonstrated an acute but transient net increase of a subset of Ser^473^ Akt1 forms in response to PI3K inhibition (Fig. 5, *B* and *D*). Conversely, Thr^308^ phosphorylation was consistently and homogenously removed from all Akt forms with a similar time course. Perhaps the most compelling evidence for inherent heterogeneity in the Ser^473^/Thr^308^-phosphorylated Akt species that are present in neurons came from our comparative analysis of growth factor and PTEN deletion experiments (Figs. 8 and 9). We were able to observe differential sensitivities of certain Ser^473^-Akt species to PTEN deletion. In this respect, the acidic Akt1 forms appeared to be more sensitive. It has to be noted that deletion of PTEN is supposed to impact primarily on the plasma membrane pool of PIP_3_ (36) and only secondarily on the plasma membrane or intracellular pools of phosphatidylinositol 3,4-bisphosphate (37). Intriguingly, a recent study suggested that Akt2 but not Akt1 can be activated on endosomal membranes by insulin by a pathway involving a class II PI3K and the localized production of phosphatidylinositol 3,4-bisphosphate (38). Similarly, when compared with PTEN deletion, different Ser^473^/Thr^308^-Akt species were generated by growth factor treatments. In this case, the more basic Ser^473^/Thr^308^-Akt1/2 species were primarily regulated by almost all growth factors tested, namely BDNF, EGF, and insulin, albeit to different degrees. These studies show unequivocally that although Akt can be regulated by both growth factors and PTEN, these two pathways do not result in the generation of the same Ser^473^/Thr^308^ Akt species. Indeed, our current studies provide the first—to our knowledge—molecular evidence that PTEN-deficient cells (neurons) have a distinct molecular signature compared with growth factor-stimulated cells when it comes to downstream Akt phosphorylation. In previous studies on the dependence of PTEN-deficient tumors on individual Akt isoforms, it has been reported that Akt1 and Akt2 have opposing roles on tumorigenesis of PTEN knock-out astrocytes and that total Akt phosphorylation is not predictive in this setting (39). Furthermore, in recent years, the dependence of PTEN-deficient cells on upstream PI3K isoforms has gained considerable attention, particularly in the cancer field. The consensus appears to be that PTEN-deficient tumors are more sensitive to p110β and, depending on the genetic background, also p110α isoforms of class I PI3Ks (40–42). In our unpublished studies, we have asked whether inhibition of individual class I PI3K isoforms produce a different Akt phosphorylation signature in wild-type and PTEN-deficient neurons. Interestingly, in both cases, the predominant isoform contributing to Akt phosphorylation is p110α, and thus far, we were unable to detect any differential impact of p110β, γ, or δ isoform inhibition.^5^ Whether these observations reflect Akt isoform specificities or differences in the developmental states of neurons remains an important question to be addressed.

In addition to the differences between Ser^473^ and Thr^308^ Akt phosphorylation discussed above, we observed a major discrepancy in the distribution of Ser(P)^473^- and Thr(P)^308^-recognized peaks in rat brain lysates. We were able to conclude that the new Thr^308^ peaks with basic pIs corresponded to phosphorylated PKC and not Akt forms. These peaks were also observed in primary cortical neurons in low amounts, but surprisingly, they represented the vast majority of Thr^308^ antibody-detected signal in aging rat brain lysates. Whether this result is merely due to an increased abundance of classic PKC isoforms α, β, and γ, combined with cross-reactivity of the Thr^308^-Akt antibody toward the equivalent PDK1-dependent PKC phosphorylation site (43, 44) or whether it reflects a true progressive shift of PDK1 regulatory phosphorylation from Akt toward PKCs during postnatal development and aging warrants further study.

Analysis of the Akt profiles during postnatal brain development revealed interesting peak shifts during the first weeks of postnatal life. Our results of Akt, GSK3β, and ERK1/2 expression and phosphorylation during postnatal rat brain development are in accordance with previous results for mouse hippocampal and cortical development (45). Akt3 has been proposed to play an essential role in postnatal brain development, and we detected a decreased expression of Akt3 after 3 weeks (46). A peak in Akt2 expression 3 weeks after birth may be suggestive of a specific role in the non-neuronal cell development at this time point (47). Postnatal brain development is characterized by dramatic changes in the cellular composition of the brain. During the first week, the net number of neurons increase dramatically followed by an increase in non-neuronal cells in postnatal weeks 2 and 3 (47). The changes in Ser^473^ and pan-Akt profiles were most evident when comparing the P0 and P7 samples (Fig. 6, *B* and *D*, *top two panels*). This suggests a substantial reprogramming of Akt phosphorylation and activation during these crucial developmental stages.

In summary, our work identifies differential signaling of the three Akt isoforms in neuronal cells and tissue. By employing cIEF in a neuronal background, we were able to demonstrate activation of different isoforms by different signaling cues, as well as isoform-specific expression patterns during postnatal brain development. This study provides a first step to decipher the complex phosphorylation and activation events of Akt in a time- and signal-dependent manner in neurons.

## Author Contributions

S. S. and G. L. performed the experiments. All authors analyzed the results and wrote the manuscript.

## Footnotes

5 S. Schrötter, G. Leondaritis, and B. J. Eickholt, unpublished data.

## Footnotes

4 The abbreviations used are:

PIP_3_: phosphatidylinositol 3,4,5-trisphosphate
cIEF: capillary-based isoelectric focusing
pI: isoelectric point
GSK: glycogen synthase kinase
mTOR: mammalian target of rapamycin
WM: wortmannin
TPA: tetradecanoyl-phorbol-acetate
Bicine: *N*,*N*-bis(2-hydroxyethyl)glycine
BDNF: brain-derived neurotrophic factor
DIV: days *in vitro*
PTM: post-translational modification
WB: Western blotting
P*n*: postnatal day *n*.
